# Thermally Drawn Elastomer Nanocomposites for Soft Mechanical Sensors

**DOI:** 10.1002/advs.202207573

**Published:** 2023-02-28

**Authors:** Andreas Leber, Stella Laperrousaz, Yunpeng Qu, Chaoqun Dong, Inès Richard, Fabien Sorin

**Affiliations:** ^1^ Institute of Materials École polytechnique fédérale de Lausanne Lausanne 1015 Switzerland

**Keywords:** conductive polymer nanocomposites, functional fibers, pressure sensors, soft materials, soft robotics, strain sensors

## Abstract

Stretchable and conductive nanocomposites are emerging as important constituents of soft mechanical sensors for health monitoring, human–machine interactions, and soft robotics. However, tuning the materials’ properties and sensor structures to the targeted mode and range of mechanical stimulation is limited by current fabrication approaches, particularly in scalable polymer melt techniques. Here, thermoplastic elastomer‐based nanocomposites are engineered and novel rheological requirements are proposed for their compatibility with fiber processing technologies, yielding meters‐long, soft, and highly versatile stretchable fiber devices. Based on microstructural changes in the nanofiller arrangement, the resistivity of the nanocomposite is tailored in its final device architecture across an entire order of magnitude as well as its sensitivity to strain via tuning thermal drawing processing parameters alone. Moreover, the prescribed electrical properties are coupled with suitable device designs and several fiber‐based sensors are proposed aimed at specific types of deformations: i) a robotic fiber with an integrated bending mechanism where changes as small as 5° are monitored by piezoresistive nanocomposite elements, ii) a pressure‐sensing fiber based on a geometrically controlled resistive signal that responds with a sub‐newton resolution to changes in pressing forces, and iii) a strain‐sensing fiber that tracks changes in capacitance up to 100% elongation.

## Graphical abstract

Thermoplastic elastomer‐based nanocomposites, with specifically engineered rheological, mechanical, and electrical characteristics, enable multimodal mechanical sensing in thermally drawn fibers. Taking advantage of the materials’ properties and the design freedom of the thermal drawing process, the fiber‐based strain sensors can be tailored toward specific modes of deformation as well as integrate additional functionalities, such as actuation and light delivery.

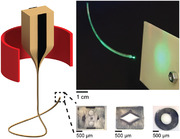

## 1 Introduction

Soft electromechanical sensors have been at the heart of key developments in numerous application fields, including health and sports monitoring,^[^ ^1^, ^2^ ^]^ human–machine interaction,^[^ ^3^, ^4^ ^]^ and soft robotics.^[^ ^5^, ^6^ ^]^ In such applications, a mechanical stimulation, in the form of an elongation, compression, bend or torsion that occurs across different ranges of magnitudes, is effectively converted into an electrical signal. Several signal generation mechanisms have been proposed, typically based on controlled variations of resistance or capacitance.^[^ ^7^ ^]^ Regardless of mechanisms, a soft electromechanical sensor must comprise suitable materials and device architectures to be able to deform to large extents, be receptive toward the desired mode of stimulation, and exhibit a sensitivity tuned to the targeted loading intensity.

To achieve both mechanical deformability and electrical conductivity in a device material, researchers often rely on nanocomposites. In these materials systems, conductive nanofillers, such as carbon nanotubes (CNTs),^[^ ^8^ ^]^ graphene,^[^ ^9^ ^]^ carbon black,^[^ ^10^ ^]^ or metallic nanowires,^[^ ^11^, ^12^ ^]^ are dispersed within an elastomeric matrix, such as polydimethylsiloxane or thermoplastic elastomers,^[^ ^13^, ^14^, ^15^, ^16^ ^]^ forming a conductive pathway through a percolated network. In addition to the materials, the device fabrication approach, with which conductive nanocomposites are arranged with standard elastomer dielectrics to realize the desired 3D device architectures, plays an important role. Proposed manufacturing approaches for thermoset‐based systems include nanomaterial deposition,^[^ ^17^ ^]^ molding,^[^ ^18^ ^]^ printing^[^ ^19^, ^20^ ^]^ and wet spinning.^[^ ^21^ ^]^ However, these techniques yield devices that are typically limited in size, structural complexity, and manufacturing throughput.

Nanocomposites based on thermoplastic elastomers rather than standard thermoset elastomers provide a unique opportunity in this regard, because this polymer class is in principle compatible with polymer melt processing techniques, such as injection molding,^[^ ^22^ ^]^ compression molding,^[^ ^23^ ^]^ extrusion and melt‐spinning,^[^ ^15^, ^24^, ^25^ ^]^ and 3D printing.^[^ ^26^ ^]^ This family of techniques relies on the viscous flow of polymer melt under simultaneous application of heat and stress, and yields complex 3D products at high throughputs. The thermal drawing process, in particular, stands out because it enables the scalable manufacturing of fibers that integrate multiple materials arranged in fine cross‐sectional architectures with µm‐scale feature sizes across extended lengths of 10s of meters.^[^ ^27^, ^28^, ^29^, ^30^, ^31^, ^32^ ^]^ While this is an attractive prospect, in practice it is difficult to process thermoplastic elastomer‐based nanocomposites by thermal drawing or other viscous polymer melt‐based techniques. The reduced processability of nanocomposites is related to the percolated network of nanofillers that introduces a significant melt elasticity and yield behavior, which impede the controlled viscous flow of material.^[^ ^33^ ^]^ Moreover, the manufacturing process heavily influences the conductivity of the nanocomposites,^[^ ^34^ ^]^ altering the performance of the resulting sensor device, especially for high draw ratio (i.e., ratio of preform to fiber diameters).^[^ ^15^ ^]^ Because of these manufacturing challenges, previously proposed soft sensors integrating thermoplastic elastomer‐based nanocomposites have suffered from rudimentary device architectures and limited capabilities.

In this article, thanks to an in‐depth rheological analysis, we enable the processing of soft nanocomposites via the preform‐to‐fiber thermal drawing technique to realize advanced electronic fiber sensors. Based on this analysis, we purposefully engineer a nanocomposite consisting of carbon nanotubes dispersed in a thermoplastic elastomer‐based matrix. By combining the conductive nanocomposite with a dielectric cladding, we obtain multimaterial fibers that are simultaneously mechanically stretchable and electrically conductive. Moreover, through an adaption of the thermal drawing process, we achieve stable drawing for an extended range of process temperatures, allowing the tailoring of the nanocomposite conductivity as well as strain‐sensing sensitivity and range. At a lower processing temperature, the sensitivity in the resulting fibers is significant enough to capture compressive strains below 1%, while at elevated temperatures, we achieved a 15‐fold increase in conductivity compared to the low‐temperature‐fiber and such conduction can be maintained for stretching up to almost 200%. Based on the materials and processing platform, we develop a diverse set of fiber‐based mechanical sensors with complex cross‐sectional structures and fabricated at large scale. We program the piezoresistive sensitivity of the nanocomposites through the fabrication process and integrate them in fiber architectures targeted at specific modes of stimulation. We demonstrate a high degree of design freedom and application potential through i) a robotic fiber where an integrated bending mechanism is monitored by a piezoresistive feedback, ii) a pressure‐sensing fiber based on geometrically induced variations of resistance, and iii) a stretch‐sensing fiber based on measures of capacitance.

## 2 Results and Discussion

### 2.1 Thermal Drawing Fabrication Process and Rheological Analysis of Nanocomposites

**Figure 1:**
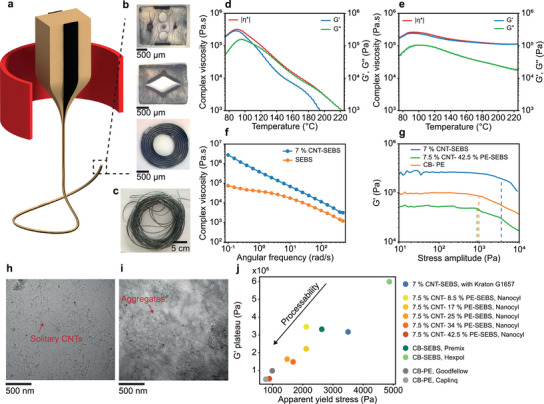
Materials, fabrication, and rheological investigation of nanocomposites. a) Schematic of the thermal drawing process. b) Optical images of fiber cross‐section. Each fiber architecture is targeted at specific modes of stimulation such as bending, pressure or stretch sensing. c) Photograph of a fiber of length 10 m to illustrate the scalability of the process. d) Temperature ramp experiment of the thermoplastic elastomer SEBS. A crossing point is observed at ≈110 °C, above which the material behavior is dominated by viscous flow. e) Temperature ramp experiment of the nanocomposite 7 wt% CNT–SEBS. The storage modulus remains larger than the loss modulus for all the temperatures in the range of interest, suggesting a pronounced melt elasticity. f) Frequency sweep experiments at 140 °C for both pure SEBS and 7 wt% CNT–SEBS nanocomposite. The near‐linear relationship in the logarithmic scale between the complex viscosity and the frequency for the nanocomposite is indicative of a pronounced melt elasticity. g) Storage modulus as a function of the oscillation stress amplitude for different conductive composite systems. The onset of the curve indicates the start of irreversible plastic deformation, also defined as the apparent yield stress. h) Transmission electron image of 7 wt% CNT–SEBS. i) Transmission electron image of 7.5 wt% CNT‐ 42.5 wt% PE–SEBS. j) Nanocomposite selection map, quantified by the storage modulus plateau value and the apparent yield stress. The arrow pointing toward lower moduli and apparent yield stresses indicates the improved processability by thermal drawing.

To fabricate the fiber‐based sensors, we seek to employ the thermal drawing process. This technique involves the conversion of a macroscopic preform to a thin and long fiber through controlled viscous flow of material under simultaneous application of heat and tension (**Figure** **1**a). The preform is readily prepared using standard thermoplastic processing techniques, including the shaping of individual material parts by compression molding or mechanical machining, and the subsequent assembly and consolidation of the parts in a final compression molding step. During the thermal drawing process, the preform is essentially elongated to extreme extents, and thus the cross‐sectional structure is preserved from preform to fiber, at typical scale‐down factors of 10 to 30 respective to the diameter. We demonstrate the design freedom of the technique by fabricating several fibers integrating a soft nanocomposite in a thermoplastic elastomer cladding (Figure 1b), each envisioned as mechanical sensors for a specific mode of stimulation, as will be discussed later. The advantage of the process is that such complex multimaterial structures with feature sizes on the order of micrometers are realized over 10s of meters of fiber length (Figure 1c). However, as other thermoplastic fabrication techniques, thermal drawing is also restrictive in the materials that can be processed. Recently, it was found that compatible polymers exhibit a specific rheological profile in an oscillatory shear rheology experiment.^[^ ^35^ ^]^ We show this behavior for the thermoplastic elastomer poly[styrene‐*b*‐(ethylene‐*co*‐butylene)‐*b*‐styrene] (SEBS) in Figure 1d. As the temperature is increased, the material transitions from a mainly elastic state, where the storage modulus *G*′ is significantly higher than the loss modulus *G*″, to a viscous state where *G*″ dominates. The crossover of *G*′ and *G*″ at 110 °C, which corresponds to the glass transition temperature of the hard polystyrene segment in the copolymer, delimitates the onset of the processing temperature window. Although both moduli decrease as the temperature is increased further, *G*″ remains at an elevated level, resulting in a high viscosity value of 10^4^ to 10^5^ Pa s in the processing window. Indeed, it is this pronounced viscosity that supports the stresses during the drawing process and retains the structural integrity of the fiber, which is impaired in low‐viscosity materials by thermal reflow.

To impart electrical conductivity to a material that is thermally drawable and mechanically deformable, we first introduce CNTs in the same grade of SEBS through melt mixing. By dilution of a masterbatch, we set the filler concentration to 7 wt%, at which we find the material to be sufficiently conductive (*ρ* = 0.1 Ω m). The CNT content and distribution indeed have a strong influence on the nanocomposite electrical conductivity and sensitivity. Increasing the CNTs loading leads to an improved electrical conductivity, but also a reduced sensitivity. Indeed, a denser CNT network is accompanied by a lower electromechanical coupling as it will be explained later in this paper. In this study, the CNT concentration was fixed to 7 wt% to meet a tradeoff between conductivity, sensitivity, and processability. The resulting nanocomposite exhibits a rheological behavior that is dramatically different from the polymer base material SEBS (Figure 1e). Although the magnitude of the viscosity ranges on the same order (|η*| = 1.6 × 10^5^ Pa.s for SEBS and |η*| = 2.4 × 10^5^ Pa.s for 7 wt% CNT–SEBS at 110 °C), the nanocomposite is dominated by an elastic rather than viscous behavior, underlining the importance of employing oscillatory rather than continuous rheology. In fact, no crossover of *G*′ and *G*″ is observed, and *G*′ remains the larger of the two dimensions and is virtually unchanged by an increasing temperature. While this rheological behavior does exclude CNT–SEBS from being individually processed by thermal drawing, it can still possibly be codrawn with a stress‐supporting material that features a viscous nature counteracting the elasticity of the nanocomposite. For instance, carbon black‐reinforced polymers have been drawn and exploited for their electrical properties.^[^ ^36^, ^37^ ^]^ Carbon black‐reinforced polyethylene (CB‐PE), a conductive but hard nanocomposite that exhibits a very similar rheological profile to CNT–SEBS (Figure S1, Supporting Information), could be drawn within supporting claddings of polycarbonate^[^ ^38^ ^]^ as well as SEBS.^[^ ^27^ ^]^ However, drawing experiments of our nanocomposite CNT–SEBS supported by pure SEBS were unsuccessful (Figure S2, Supporting Information).

Because previous thermal drawing criteria are unsuitable, we need to establish a rheological assessment adapted to nanocomposites, based on an understanding of the dynamic microstructure. In composite systems with elevated filler concentrations, the filler–filler interactions result in the formation of a physical network, often termed rheological percolation, which restrains the long‐range motion of polymer chains.^[^ ^39^, ^40^ ^]^ This network structure significantly contributes to melt elasticity, resulting in a change from a viscous fluid to solid‐like behavior.^[^ ^41^ ^]^ This behavior is confirmed in a rheological frequency sweep, where the nanocomposite exhibits a linear behavior in the log–log scale at all frequencies, whereas the pure polymer enters a Newtonian region at low frequencies, as we show in Figure 1f. Such a linear behavior is usually accompanied by a high storage modulus and the appearance of a pronounced yield stress.^[^ ^40^ ^]^ Both of these characteristics are known to adversely affect the processability of melts, manifested for instance by a reduced maximum attainable melt draw ratio in melt spinning.^[^ ^41^ ^]^ To investigate this effect, we seek to quantify the storage modulus and the yield stress of our nanocomposite through a stress amplitude sweep in oscillatory shear rheology (Figure 1g). We carried out this test at 140 °C, which is the ideal thermal drawing temperature of pure SEBS. Note that, in any case, beyond the softening point of the matrix, the temperature has a minor influence on rheological percolated nanocomposites (Figure S3, Supporting Information). In the test, the onset of decrease of the *G*′ curve marks the apparent yield stress, at which point irreversible plastic deformation occurs. The details of the extraction of the apparent yield stress are shown in Figure S4 of the Supporting Information. Indeed, we find a high storage modulus plateau of 330 kPa and an apparent yield stress of 3.5 kPa for CNT–SEBS, in contrast to the drawable reference material CB‐PE with a storage modulus plateau of 100 kPa and an apparent yield stress 1 kPa. To explain this behavior, we employ transmission electron microscopy to image our nanocomposites, because the elastic characteristics in nanocomposites are highly dependent on the network morphology. Figure 1h was taken at a particular cross‐section yet is representative of what we could observe along the fiber length. We find CNTs to be in a highly dispersed state with little visible agglomeration, a configuration that indeed is known to contribute to a pronounced melt elasticity.^[^ ^42^ ^]^

Guided by this characterization, we adapted our nanocomposite to a ternary system with 7.5 wt% CNTs, 42.5 wt% PE, and 50 wt% SEBS, which we fabricated by diluting a CNT–PE masterbatch with SEBS. The reasoning behind our approach is to break the distributed percolated network of fillers, into percolated regions of conducting composites. That way, electrical conductivity is preserved but the effect of a distributed network of fillers on the thermomechanical properties is drastically reduced. In this nanocomposite, we find the CNTs to be less dispersed and predominantly in the form of interconnected clusters (Figure 1i). It has been reported by Alig et al. that a certain degree of agglomeration is beneficial to the nanocomposite conductivity, and that networks with well dispersed CNTs were, in some cases, found to be electrically insulating.^[^ ^34^ ^]^ The microstructure of the CNT–PE–SEBS nanocomposites studied in this paper seems to meet the required criteria in term of CNTs concentration and dispersion to lead to a stretchable and conductive composite which can be integrated into thermally drawn fibers.

This changed microstructure is reflected in the rheological properties (Figure 1g), specifically by a reduced storage modulus and yield stress. In an analysis of the effect of PE content in the nanocomposite, we find a proportional reduction of elastic characteristics. A detailed rheological analysis of the different nanocomposite formulations is shown in Figure S5 of the Supporting Information.

Indeed, when integrated in an SEBS cladding, this nanocomposite could be successfully thermally drawn (Figure 1c). The nanocomposite microstructure after thermal drawing is shown in Figure S6 of the Supporting Information. We summarize our findings in a materials selection map, with which nanocomposites compatible with thermal drawing can be identified through the key parameters of plateau storage modulus and apparent yield stress. We also include several other materials systems that we trialed during our research (Figure 1j), encompassing different grades of SEBS as matrix materials as well as carbon black as an alternate nanofiller. Through thermal drawing tests of all the considered nanocomposites, we find that successful processing scales directly with lower plateau storage modulus and apparent yield stress.

### 2.2 Mechanical and Electrical Properties of the Nanocomposite Fibers

**Figure 2:**
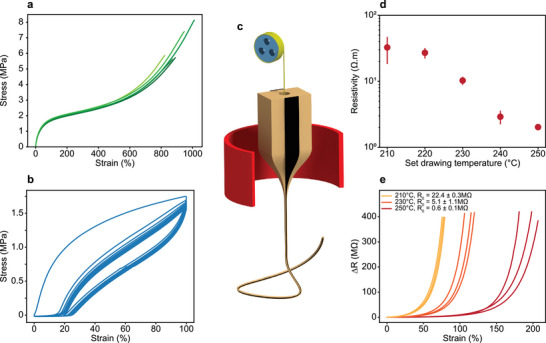
Mechanical and electrical characterization of the nanocomposite fibers. a) Static tensile tests of fibers composed of 7.5 wt% CNT‐ 42.5 wt% PE–SEBS in an SEBS cladding. The curves correspond to multiple fiber samples. b) Dynamic tensile test of a nanocomposite fiber cyclically stretched between 0 and 100% strain for 10 cycles. c) Schematic of the adapted thermal drawing process. A draw‐supporting 1D element, termed spine, is introduced to extend the range of processing temperatures. d) Nanocomposite resistivity in the fiber for different set drawing temperatures. The error bar represents the standard deviation. e) Piezoresistivity of nanocomposite fibers drawn at different temperatures, where fibers processed at higher temperatures exhibit a reduced sensitivity to deformation and increased strain limit of the electrical conduction breakdown.

We address next the mechanical and electrical properties of the fabricated fibers, which are crucial for mechanical sensing applications. In our mechanical analysis, we focus on thermally drawn fibers, composed of the nanocomposite 7.5 wt% CNT‐ 42.5 wt% PE–SEBS embedded in an SEBS cladding. A characterization of the individual materials is detailed in Figure S7 of the Supporting Information. In static tensile tests of the fibers (**Figure** **2**a), we measure a modulus at 100% strain of ≈1.74 ± 0.04 MPa and an elongation at break of 908% ± 65%. At the terminal elongation, both material components of the fibers fail simultaneously, indicating a strong interfacial bonding. We also submitted the fibers to dynamic tensile tests, where the strain was varied between 0% and 100% for 10 cycles (Figure 2b). The materials’ response was predominantly elastic, where the fibers return to their initial configurations as the load is removed. However, a hysteresis and strain softening behavior is recorded, which is often observed for elastomeric systems and is related to the viscoelastic nature of the materials. We quantify the stretchability of the fibers by a remnant elongation when the load returns to zero after 100%‐stretching. The remnant elongation was 20% after 1 cycle. In subsequent cycles, only minor changes of the hysteresis curve were observed. Thus, in applications, a prestretching can be introduced to assure that the fiber remains entirely elastic and taut within the targeted strain range. As a conclusion of the mechanical analysis, we find that the softness and elastic stretchability qualify the nanocomposite fibers for applications in soft electronics and wearable devices.

We investigate the electrical properties by measuring the resistivity of the nanocomposite in fibers (see the Experimental Section for details). While the nanocomposite is indeed conductive, we find the resistivity can increase to up to 32.7 Ω m for fibers drawn at low temperature, compared to the predrawing value of 0.1 Ω m, as shown in Figure 2d. This effect is caused by the restructuring of the conductive filler network under the severe deformation of the thermal drawing process.^[^ ^34^, ^43^ ^]^ It is known to be strongly dependent on the processing conditions, most importantly the temperature as it alters significantly the viscosity of the polymer and thus the shear forces acting on the filler aggregates.^[^ ^44^ ^]^ As presented in Figure 1d, above the crossover temperature, the complex viscosity of SEBS rapidly decreases with increasing temperature, and transitions from 1.6 × 10^5^ Pa s at 110 °C to 1.5 × 10^4^ Pa s at 160 °C. Therefore, by increasing the drawing temperature, stresses transferred to the filler network may be lower, reducing the probability of conductive pathways destruction, and therefore preserving the network integrity. However, for thermal drawing, the processing temperature window is narrow, because the reduced viscosity of the fiber materials under increased temperature can lead to uncontrolled flow and fiber breakage. Thus, to extend the processing temperature range toward higher values, we adapt our process and introduce a rigid spine as a flow‐controlling element (Figure 2c). The spine is an inert 1D element, of diameter 50 to 760 µm and in the form of a solid metal wire or polymer line, that is introduced into a dedicated channel in the preform before the process starts. As the materials of the preform begin to flow into the fiber, the channel narrows, latching on to the spine and unspooling it continuously as the fiber is drawn. Note that the spine can additionally play a functional role in the produced fiber, such as electrical or optical guiding, or be removed in postprocessing to render the fiber stretchable again, as will be shown later. The process extension allows us to increase the upper temperature limit by 40 °C, resulting in a drawing range of 210–250 °C. Note that these temperatures represent the set machine temperatures, which do correspond, but are not equal to the true temperatures experienced by the material (the temperature range experienced by the materials is estimated at 135–160 °C). By drawing nanocomposite fibers at different temperatures, we record a strong variation in resistivity (Figure 2d) of the composite after drawing. At the highest processing temperature, we achieve a resistivity of 2.0 Ω m, which is lower by a factor of 15 compared to the low‐temperature value. As we explained above, the effect of reducing the viscosity and shear rate experienced by the composite is clearly exhibited it.^[^ ^43^ ^]^ Thus, through the processing temperature alone, we can effectively tune the resistivity of the nanocomposite over almost 2 orders of magnitude.

We also assessed the effect of processing temperature on the piezoresistivity, which is known to be closely linked to the resistivity for composite systems.^[^ ^45^ ^]^ By measuring the change in resistance during elongation of the fibers (Figure 2e), it is first striking to see a shift in behavior as the strain increases. At low strain, the percolation paths are marginally affected and the increase of resistance is due to the change of geometry of the sensor as it elongates in one direction and shrinks in the cross‐section. As the strain increases, percolation paths disconnect and the resistivity increases as well, leading to a faster change in resistance. As we can see in Figure 2e, we find that the nanocomposites processed at higher temperature respond less sensitively to deformation at low strain, as indicated by a smaller slope of the resistance–strain curve (gauge factor at 70% strain of 94.2 ± 11.4 at 210 °C, 38.6 ± 1.8 at 230 °C, and 18.6 ± 0.5 at 250 °C, for details refer to Figure S8, Supporting Information). This difference is a direct result of the higher number of percolation paths that remain after drawing at high temperature as explained above, leading to a larger strain window over which the resistance change is due to geometry only. Additionally, the electrical conduction breaks down (when the applied current deviates from the set current by more than 50%) at significantly higher strains for higher processing temperatures (77% strain at 210 °C and 195% strain at 250 °C). The piezoresistivity effect of nanocomposites is governed by the conductive network connectivity: both the resistance and the number of single conductive paths determine the final electrical resistance of the material. By stretching the fiber, the interparticles separation grows, increasing the tunneling resistance and potentially leading to complete loss of connection between aggregates at higher strains.^[^ ^46^, ^47^ ^]^ These effects are expected to be less pronounced for denser conductive networks obtained at higher drawing temperatures, explaining the lower sensitivity to deformation observed for nanocomposite fibers drawn at higher temperatures.^[^ ^25^ ^]^

Note that along with the ability to realize complex multimaterial structures in long fibers, the tunable electrical properties of the integrated nanocomposites promote the thermal drawing process to a versatile platform for the fabrication of sensors that can be targeted at specific modes and ranges of mechanical stimulation.

### 2.3 Piezoresistive Bending Feedback in Soft Robotic Fibers

**Figure 3:**
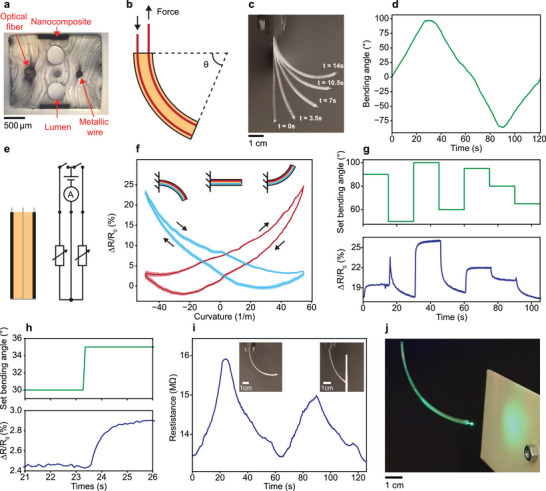
Piezoresistive feedback in robotic fibers. a) Optical image of a robotic fiber cross‐section, integrating two nanocomposite sheets, a metallic wire, an optical guide, and two lumens that can host tendons to actuate the fiber. b) Schematic of the fiber bending controlled by integrated tendons and quantified by the bending angle *θ*. c) Sequence of overlaid photographs of the fiber during a bending ramp experiment. d) Bending angle, quantified by a camera and computer vision, as a function of time. e) Schematic of the side view of a fiber with two nanocomposite electrodes, highlighted in black, and a metallic wire in gray (left), and the resulting equivalent electrical circuit (right). f) Relative resistance change versus the fiber curvature for both nanocomposite electrodes. The color coding in the schematics indicates the state of tension or compression for the two curves. The line represents the mean and the shaded area indicates the standard deviation for 10 bending cycles. g) Resistive response of the piezoresistive nanocomposite sheet for a sequence of arbitrarily set bending angles. h) Piezoresistive response of the nanocomposite to a small bending angle step of 5°. i) Resistive response of the nanocomposite during two cycles of bending. In the first cycle, the bending unopposed, whereas, in the second cycle, the motion is hindered by an obstacle, altering the electrical signal. j) Robotic fiber emitting light through the integrated optical guide onto a screen.

Having assessed the rheological, mechanical, and electrical properties, we now integrate the soft nanocomposite in functional fibers for sensing applications. In our first device example, we take advantage of the material's strain‐sensitive resistance to monitor the extent of fiber bending induced by an embedded actuation mechanism. The rectangular cross‐section of the robotic fiber, which integrates nanocomposite sheets, lumens, an optical guide, and a metallic wire in an SEBS cladding, is shown in **Figure** **3**a. The fiber bending mechanism is driven by tendons, which take the form of nylon lines of diameter 200 µm introduced in the two lumens. By fixing the tendons at the distal end and concertedly pulling on them at the proximal end, the fiber bends to controlled amounts quantified by the bending angle (Figure 3b). We test this actuation mechanism by applying a bending angle ramp from 0° to 90° and back for both bending directions and tracking the movement with computer vision (Figure 3c,d). Indeed, the configuration of the fiber can be precisely controlled with the integrated tendons. Next, we aim at generating a feedback signal with the integrated nanocomposites to enhance the controls of the robotic fiber, because configurations of soft robotic systems are often difficult to predict based on input alone, particularly in dynamic environments where obstacles can be encountered. However, with a cross‐sectional height of 1.7 mm, the strains induced by the bend in the thin fiber scale below 1.3% for the targeted bending angle range of 0° to 90° and a fiber length of 100 mm. To capture such low levels of strains, we draw the fibers at the lowest processing temperature, which results in the highest resistivity and piezoresistivity of the incorporated nanocomposites. Moreover, we position the nanocomposite sheets on the outer faces of the fibers because the highest bending‐induced strains are experienced at the position furthest from the neutral plane.^[^ ^48^ ^]^ To interrogate the two nanocomposite sheets selectively at the proximal end, we connect the two sheets at the distal end to the embedded metallic wire, which acts as a grounding element (Figure 3e).

The resistive responses of the nanocomposites are shown in Figure 3f for a complete cycle of bending of −90° to 90°, where the sign of the bending angle indicates the bending direction. For a given bending angle, one of the nanocomposite sheets is in tension while the opposing one is in compression. As shown, in compression, the resistance increases significantly up to a relative change of 25%, whereas, in tension, only a small nonmonotonic response below 3% is recorded. Since the observed increase of resistance in compression is opposite to the expected decrease due to the change in geometry, the resistive response must be dominated by the piezoresistive effect. In nanocomposites, the piezoresistivity results from the destruction and formation of conductive pathways and is highly dependent in trend direction and magnitude on the morphology of the percolated nanofiller network.^[^ ^46^ ^]^ Practically, the disparate resistance variations in compression and tension enable the identification of bending direction, and the pronounced response in compression allows the estimation of bending angle magnitude. However, the bending sensor performance is limited by a hysteresis effect, which is, analogous to the mechanical properties, attributed to the viscoelastic nature of the polymer as well as the partly irreversible restructuring of the filler network during deformation. We characterized the sensor further by inducing cyclical bending with the robotic fibers in the defined range of −90° to 90° and simultaneously recording the resistive response of the nanocomposite (Figure S9, Supporting Information). After several initial cycles, the signal variation stabilizes and only a small further drift is observed, underlining the reversibility and durability of the proposed sensor. To investigate the effect of deformation history, we also induced a sequence of arbitrarily defined bending angles (Figure 3g). As shown in the temporal plot, the resistive response follows the induced bending angle, but some variations are caused by the deformation history. Nonetheless, the proposed sensor is capable of detecting angle variations as small as 5°, which is shown by the distinct resistive response to the small bending angle step (Figure 3h). Moreover, the sensor can detect obstacles on the robotic fiber trajectory. We illustrate this behavior by comparing the resistance variation through bending for an unhindered and an obstructed bending path (Figure 3i). Indeed, when an obstacle is present, the fiber motion is restrained to lower bending angles. Consequently, the maximal resistance change reached is ≈1 MΩ compared to 2 MΩ for the fiber with unrestricted trajectory. Thus, the presence of an obstacle in the path can be deduced from the resistance signal.

The results suggest that the soft robotic fibers with piezoresistive nanocomposite feedback mechanism are promising in applications that necessitate a long and thin device in unpredictable environments, such as minimally invasive interventions with active catheters and endoscopes in the biomedical field. Based on this prospect, we highlight the multifunctionality and modularity of such robotic fibers by coupling light through an integrated optical fiber (Figure 3a), which can be delivered to spatially distributed targets (Figure 3j).

### 2.4 Resistive Pressure‐Sensing Fibers

**Figure 4:**
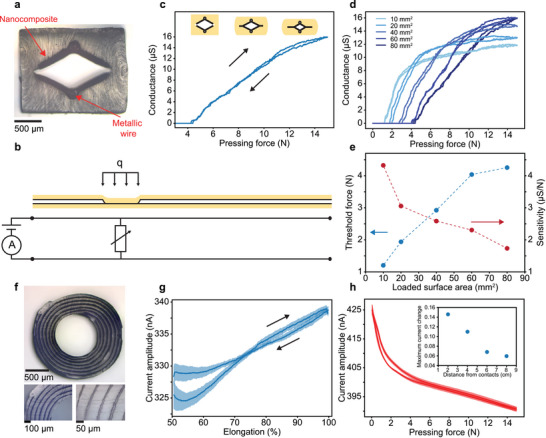
Resistive pressure‐sensing and capacitive stretch‐sensing fibers. a) Optical image of a resistive pressure‐sensing fiber cross‐section. Two nanocomposite sheets and metallic wires act as electrodes and are integrated in an SEBS support structure. b) Schematic of the fiber submitted to localized pressure and resulting equivalent circuit. c) Conductance as a function of the applied compression force for a loaded surface area of 80 mm^2^. Schematic of the fiber cross‐section during compression are shown above the graph. d) Effect of the loaded surface area. The conductance versus pressing force is recorded for different loaded surface areas. e) Threshold force and sensitivity as a function of the loaded surface area. From graph (d), the force where the nanocomposite electrodes first touch and the slope of the conductance–pressing force curve, referred as sensitivity, are reported. f) Optical image of a capacitive stretch sensing fiber. Two nanocomposite sheets (black) are rolled and act as electrodes to create a capacitive fiber with SEBS as dielectric layer. g) Stretch‐sensing performance. The current is recorded while the fiber is being stretched between 50% and 100% strain. The line represents the mean and the shaded area indicates the standard deviation for 5 cycles. h) Current flowing through the fiber as a function of the applied compression force. The line represents the mean and the shaded area indicates the standard deviation on 10 cycles. The inset shows the maximum current change $\left(\max(\right|\frac{\Delta I}{I_{0}}\left|)\right)$ for different positions with respect to the electrical contacts.

For a second fiber‐based device, we target the sensing of compressive loads, which are applied transversally on the fibers. In the proposed cross‐sectional architecture (**Figure** **4**a), sheets of nanocomposites are placed on opposing faces of a diamond‐shaped empty channel in an SEBS cladding. Additionally, Cu wires of diameter 125 µm are embedded within the nanocomposite sheets at opposing corners of the channel. In the initial configuration without load, the two electrodes, each composed of a metallic wire and nanocomposite sheet, are separated by an air gap, and thus no current can flow. As a pressure is applied on a section of the fiber, the elastic structure deforms, bringing the two nanocomposite sheets in contact and allowing a current to flow. As the wires with a resistance per length of 2 Ω m^−1^ can be neglected, the measured resistance in the equivalent circuit depends only on the nanocomposite elements and can be expressed as

(1) $$R_{m}=\frac{1}{2}\cdot \rho \cdot \frac{l}{t\cdot w}$$

where *ρ* is the nanocomposite resistivity, *l* is the length of the current pathway in the fiber cross‐section, *t* is the sheet thickness, and *w* is the width of the contact area in the fiber direction. The factor 1/2 originates from the two equal pathways the current can take through the nanocomposite sheets to travel from one wire to the other. As the compressive load is increased, the current travels a shorter distance and thus *l* decreases. The width *w*, however, is proportional to the size of the loaded area, i.e., the length of the loaded fiber segment. Thus, the measured resistance is varied based on load intensity and area at a sensitivity that is equal to the ratio of nanocomposite resistivity and sheet thickness. The equivalent circuit is shown in Figure 4b. Note that in this scheme, unlike similar previously reported fiber‐based pressure‐sensing strategies,^[^ ^27^, ^49^ ^]^ the measurement does not depend on the load's position on the fiber, because the negligible resistance of the metallic wires results in an equipotential behavior along the fiber length.

To obtain a high measurement sensitivity, we thermally draw our pressure‐sensing fibers at the lower temperature limit of the processing window, resulting in high nanocomposite resistivity. We first test the effect of load intensity, by applying a pressure ramp at constant loaded area and simultaneously monitoring the conductance (Figure 4c). As expected, at small loads, the electrodes are separated, and the conductance is zero. However, at a threshold force of 4.25 N, the conductance starts to increase, corresponding to the point of first contact. Subsequently, the conductance scales nearly linearly with the load intensity, up to the point where the signal saturates, which corresponds to the deformed configuration where the channel is fully closed, and the current pathway cannot be decreased further. In this sensing mechanism, we observe only a small hysteresis effect because structural deformation is fully reversible. Based on the results, we extract an expected pressure‐sensing sensitivity of 1.7 µS N^−1^ in the range of 4.25 to 15 N. Note that both accuracy and range can be tuned by altering the cross‐sectional geometry, for instance, through the internal angle of the diamond structure. Next, we investigate the influence of the size of the loaded area on the measured resistance, by performing pressing force ramps with indenters of increasing surface areas (Figure 4d). As a higher surface area results in lower compressive stresses, the threshold force, at which a first nonzero conductance is recorded, is shifted toward higher values. Additionally, we find that the slope of the conductance–force curve, representing the sensitivity of pressure measurement, is reduced, and the force at which the signal saturates, quantifying the sensor range, is increased for increasing loaded areas. We summarize the dependence of the threshold force and the sensitivity in a dedicated chart (Figure 4e). Finally, we also validate the durability of the proposed sensor, by submitting the fibers to 1000 pressure cycles, where we find a consistent electrical response to applied pressures with minor drifts of the signal (Figure S10, Supporting Information). We expect that the pressure sensor could be highly beneficial in large‐area electronic textiles for health monitoring or human–machine interaction, because it can readily functionalize large lengths and areas, and it responds sensitively to small changes in pressing force and load area.

### 2.5 Capacitive Stretch‐Sensing Fibers

For our final example that highlights the versatility of our platform, we enable stretch‐sensing with nanocomposite fibers based on a capacitive mechanism. The fibers integrate a rolled‐up stack of two nanocomposite sheets and two SEBS sheets that are alternately arranged, resulting in a structure resembling an axial lead capacitor (Figure 4f). By relying on this particular rolled geometry, we obtain a capacitance of 1.3 nF in the preform (Figure S11, Supporting Information), which is higher by a factor of 13 than a standard parallel plate architecture with comparable dimensions (equal electrode spacing and a width spanning the diameter). We expect this large capacitance to result in a pronounced electrical signal upon stretching. When the fiber is elongated, the electrode length increases while the electrode width and dielectric spacing decrease to a smaller extent dictated by the Poisson number, resulting in a net increase of capacitance as a function of axial strain.

For this capacitive scheme, a low resistivity and piezoresistivity of the nanocomposite is sought, which is achieved through a high thermal drawing temperature of 250 °C. We introduce a polytetrafluoroethylene tube of diameter 760 µm as draw‐supporting spine, which is mechanically removed in postprocessing. In the resulting fiber, one electrode is exposed at the inner channel and the other at the outer face, easing the connections. Note that the resulting nanocomposite sheets are of thickness 15 µm and are separated by a spacing of 80 µm, underlining the low feature sizes that can be achieved across meter‐long fibers composed of soft materials with thermal drawing. We test the electrical response to stretching by first exposing the fiber to a prestretch of 50% and subsequently elongating the fiber to 100% while monitoring the alternating current between the two capacitor electrodes (Figure 4g). As expected, the current increases as the fibers are elongated. While the behavior is reversible, a pronounced hysteresis effect at lower strains is observed. The idle times during stretch cycles induce a decrease of the current noticeable at the beginning of each cycle. Indeed, the viscoelastic nature of the material results in time‐dependent responses, particularly at high strain levels. The device is best used to sense strains between 50% and 100%. Above this strain level, the electrodes resistance could become too high and impede the device operation. Finally, we also test the suitability of the capacitive fiber as a pressure sensor, by exposing it to controlled levels of pressing forces while monitoring the current (Figure 4h). The structural deformation does indeed induce a pronounced decrease in measured current, which corresponds to a decrease in capacitance. Moreover, this effect depends on the position on the applied force, as the series resistance of the capacitor electrodes results in attenuation of the electrical current as a function of length (Figure 4h inset). We conclude that capacitive fiber can act as a capable sensor of small changes of its deformable structure, induced by stretches or pressures.

## 3 Conclusion

We have assessed the compatibility of thermoplastic elastomer‐based nanocomposites with the thermal drawing process. The microstructure of the nanocomposites, specifically the state of dispersion of the nanofillers, sensitively influences the rheological properties, such as the melt viscoelasticity and yielding behavior. However, processable materials could be identified with the key parameters of storage modulus and yield stress. Embedded in a thermoplastic elastomer cladding, the resulting nanocomposite fibers are found to be soft and stretchable (modulus of 1.74 ± 0.04 MPa and elongation at break of 908% ± 65%), as well as electrically conductive, a property that can be tuned by the processing temperature when the fiber incorporates a process‐supporting spine (32.7 Ω m at 210 °C to 2.0 Ω m at 250 °C). Along with the intricate multimaterial cross‐sectional designs, the programmable resistivity and linked piezoresistivity make the thermally drawn nanocomposite fibers a versatile platform for mechanical sensors, which can be manufactured at large scale and extreme aspect ratios. We demonstrated this potential with three fiber‐based devices, which are each targeted at specific mode of mechanical stimulation and rely on a different sensing scheme. Specifically, we developed a soft robotic fiber with an integrated bending mechanism that is monitored with a piezoresistive feedback signal; a resistive pressure‐sensing fiber that detects variations in load intensity and area through controlled structural deformations; and finally, a stretch‐sensing fiber where elongations induce reversible changes in capacitance. We expect that the proposed materials and processing platform as well as the resulting sensing concepts will significantly advance wearable devices and soft robotics.

## 4 Experimental Section

### Nanocomposites Preparation

All the nanocomposites were obtained as masterbatches (Nanocyl) and some were diluted to the desired filler concentration using a twin‐screw microcompounder (Xplore, Micro Compounder MC5) at 240 °C, 200 rotations per minute, and for 3 min. The nanocomposites were subsequently shaped using compression molding (UVL 5.0, Lauffer Pressen) at 240 °C and 1 bar for 40 min.

### Rheological Analysis

The rheological properties of the materials were assessed with a rheometer (AR 2000, TA Instruments) in a parallel plate configuration using disk samples of thickness 1 mm and diameter 25 mm. The temperature ramps were conducted from 50 to 250 °C at a ramp speed of 3 °C min^−1^, a strain amplitude of 1%, and an angular frequency of 1 rad s^−1^. The frequency sweeps were performed from 10^−1^ to 5 × 10^2^ rad s^−1^ at a temperature of 140 °C and a strain amplitude of 0.1%. For the above‐mentioned tests, all materials were characterized in the linear regime (Figure S12, Supporting Information).

In the dynamic oscillation stress sweeps, the stress amplitude was increased from 1 to 10^4^ Pa at a temperature of 140 °C (unless specified otherwise) at an angular frequency of 1 rad s^−1^. The apparent yield stress is defined as the onset value of the storage modulus curve.

### Fiber Fabrication

Individual components of the preforms were shaped from granular polymers by compression molding. Nanocomposite sheets and pure polymer parts (SEBS G1657, Kraton) were assembled and consolidated in a final compression molding step at 0.2 bar and 120 °C for 30 min. Subsequently, the preform was thermally drawn into a fiber using a custom draw tower. Additional 1D elements, including metallic wires (tin‐coated copper wires, McMaster), optical guides (optical grade plastic optical fiber unjacketed, Edmund Optics), nylon lines (clear nylon lines, McMaster), and Teflon tubing (high‐temperature tube sleeving, McMaster), were introduced using the feeding extension of the thermal drawing process. For electrical contacting, the selected conductor at the fiber end was exposed mechanically and the contact was established using silver paint for the nanocomposite and solder for integrated metallic wires.

### Microstructural, Mechanical, and Electrical Analysis

Fiber cross‐sections were imaged using optical microscopy (DM 2700 M, Leica). Samples for transmission electron microscopy were cut with an ultra‐cryomicrotome, transferred to a carbon/copper grid supports and imaged by a transmission electron microscope (F200X, Talos) at 200 kV. The mechanical properties of the nanocomposites and the fabricated fibers were assessed by uniaxial tensile testing (Z005 stand with 50 N load cell, Zwick/Roell) at an extension speed of 120 mm min^−1^. The electrical resistivity was assessed by an electrometer (Sourcemeter 2400, Keithley) at a source current of 100 nA. For the piezoresistivity assessment, fibers of 10 cm long were cut and the spine removed. The fibers were let 1 h in the clamps before each test to enable material relaxation and remove any dependence of the resistance value on the strain history. The extension speed was fixed at 5 mm min^−1^. The electrical conduction breakdown was defined as the strain where the applied current deviates from the set current by more than 50%.

### Electromechanical Analysis of Fiber Devices

For the bend‐sensing fibers, fiber samples of length 10 cm were cut. At the proximal fiber end, the composite electrodes from both fiber sides and the embedded metallic wire were contacted selectively. At the distal end, the two composite electrodes and the metallic wire were short‐circuited with silver paint. Nylon lines were inserted into the appropriate channels and mechanically fixed at the distal end, thus acting as tendons. They were used to actuate the fiber with the help of a custom setup involving two servo motors and a microcontroller, enabling a precise control of the fiber bending angle. During the test, the resistance was continuously monitored using the electrometer and the bending angles using a camera (C270, Logitech) and computer vision tracking (OpenCV in Python). For the pressure‐sensing fibers, fiber samples of length 20 to 80 cm were employed. The fibers were submitted to controlled compressive force ramps using a dynamic mechanical analysis instrument (DMA Q800, TA Instruments) in compression mode with two parallel plates at a ramp speed of 10 N min^−1^. The resistance between the two embedded metallic wires, contacted at one fiber end, was measured continuously during the test using the electrometer. For the stretch‐sensing fiber, fibers of length 10 cm were prepared, and stretched to controlled extents by a tensile testing stand (Multitest 2.5 kN, Mecmesin). Simultaneously, the alternating current between the two composite electrodes, contacted at one fiber end, was recorded using an impedance analyzer (HF2LI lock‐in amplifier, Zurich Instruments) at a voltage amplitude of 1 V and a frequency of 10 kHz.

## Conflict of Interest

The authors declare no conflict of interest.

## Supporting information

Supporting Information

## Data Availability

The data that support the findings of this study are available from the corresponding author upon reasonable request.
