# Supplementary material for: Thermally Drawn Elastomer Nanocomposites for Soft Mechanical Sensors
Source: Adv Sci (Weinh). 2023 Feb 28;10(13):2207573. doi: 10.1002/advs.202207573 (PMC10161033; doi:10.1002/advs.202207573)
Supplement: Supplementary file 1 — Supporting Information [file ADVS-10-2207573-s001.pdf]

ADVANCED SCIENCE
Open Access

Supporting Information

for *Adv. Sci.*, DOI 10.1002/advs.202207573

Thermally Drawn Elastomer Nanocomposites for Soft Mechanical Sensors

*Andreas Leber, Stella Laperrousaz, Yunpeng Qu, Chaoqun Dong, Inès Richard and Fabien Sorin**

Supporting Information

Thermally drawn elastomer nano-composites for soft mechanical sensors

*Andreas Leber[1], Stella Laperrousaz[1], Yunpeng Qu, Chaoqun Dong, Inès Richard, Fabien Sorin**

[1] These two authors contributed equally to this work.

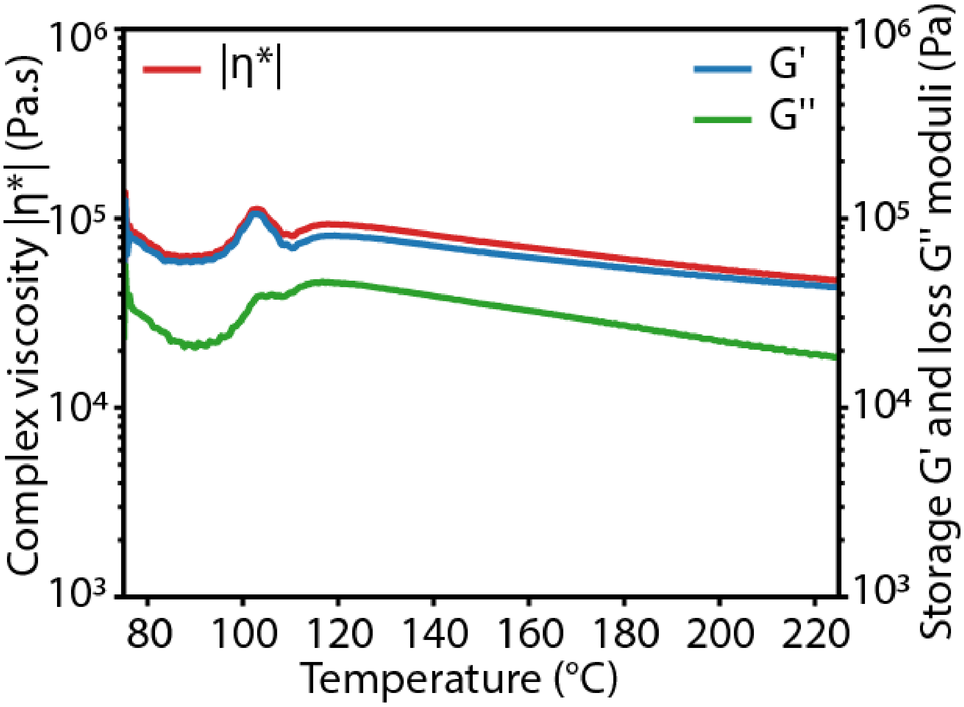

Figure S1. Temperature ramp experiment of CB-PE, Goodfellow. The temperature ramp is a useful experiment to assess the material compatibility with the thermal drawing process, and to define the processing temperature window. Ideally, around the material T_g, the loss modulus (G'') slowly changes with temperature while the storage modulus (G') decreases rapidly. A crossing point is observed, delimiting the temperature range where flow at high viscosities is expected. However, it has been shown that materials which don't obey this criterion can still be thermally drawn if associated to the adequate thermoplastic cladding. CB-PE is one of them. Indeed, as it can be seen on this figure, both the storage and loss modulus only slightly change with the temperature and no crossing point is observed. The storage modulus remains higher than the loss modulus for all temperatures in the studied range, indicating a pronounced melt elasticity characteristic of composite systems.

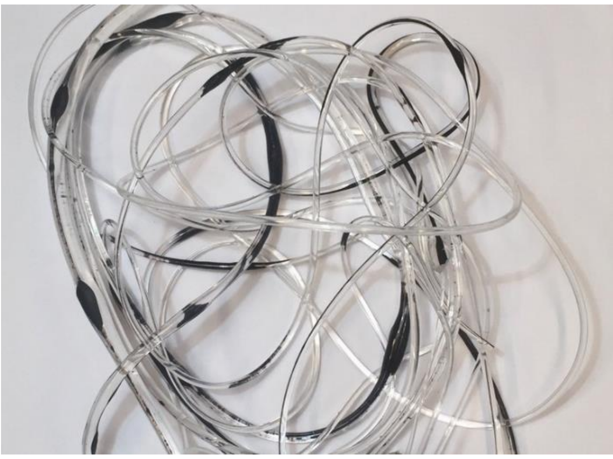

Figure S2. Fiber with 7 % CNT-SEBS composite in a SEBS cladding showing the inhomogeneous composite profile due to its pronounced melt elasticity.

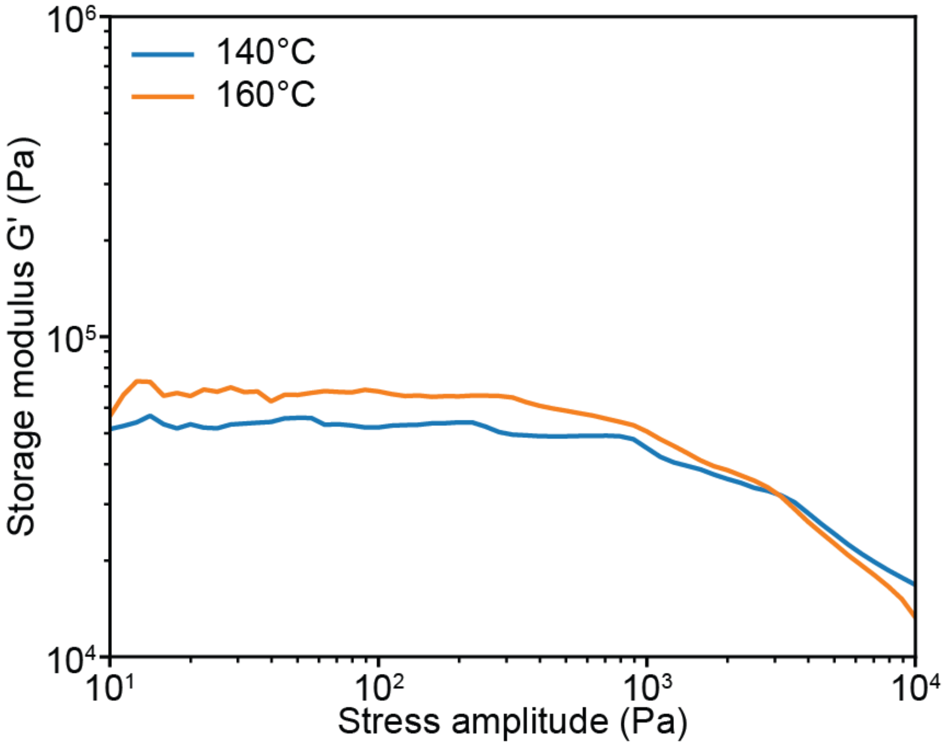

Figure S3. Storage modulus as a function of the oscillation stress amplitude of 7.5 % CNT- 42.5 % PE-SEBS nanocomposite at different temperatures. Increasing the temperature from 140 °C to 160 °C doesn't seem to significantly influence the storage modulus plateau and apparent yield stress values. Only the behavior at stress levels above the "yield point" is slightly modified, and a steeper drop of the storage modulus is observed at higher temperatures.

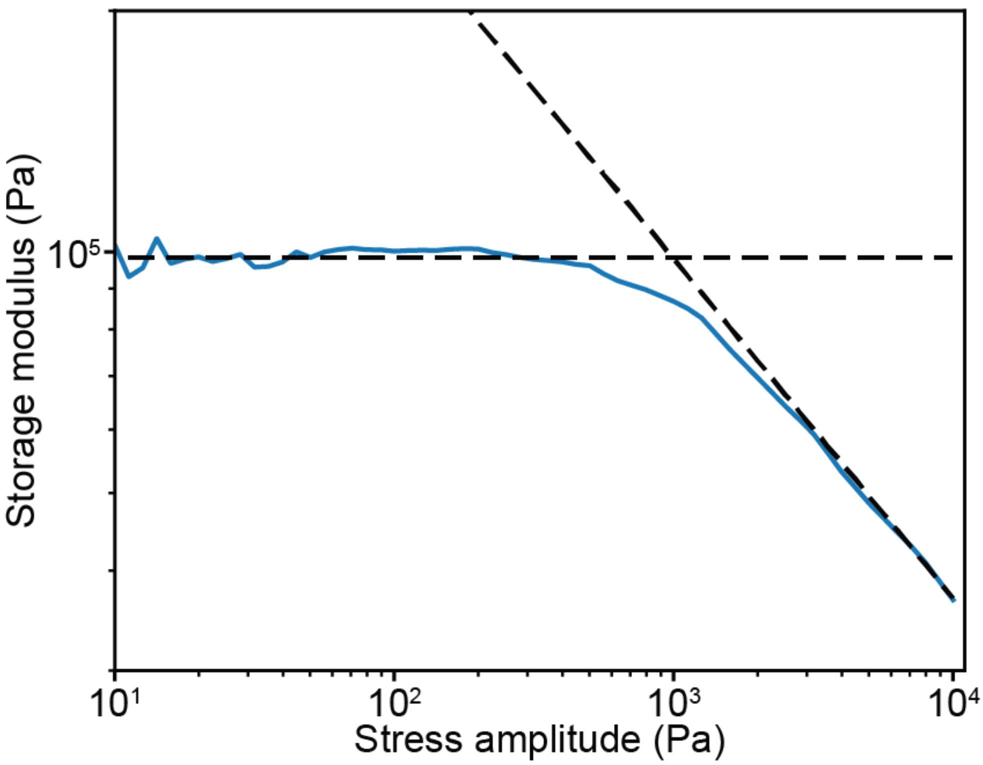

Figure S4. Yield stress extraction example (CB-PE, Goodfellow). The apparent yield stress is determined by performing a stress sweep in oscillation. The intersection between the storage modulus plateau and the linear regression of the flow curve at high stress levels (in log-log scale) enables to define the apparent yield stress value. It is important to emphasize that the value obtained through this method can't be considered as a material property strictly speaking. However, if all the test parameters are kept constant, it is a valuable way to compare different materials, and understand their flow behavior.

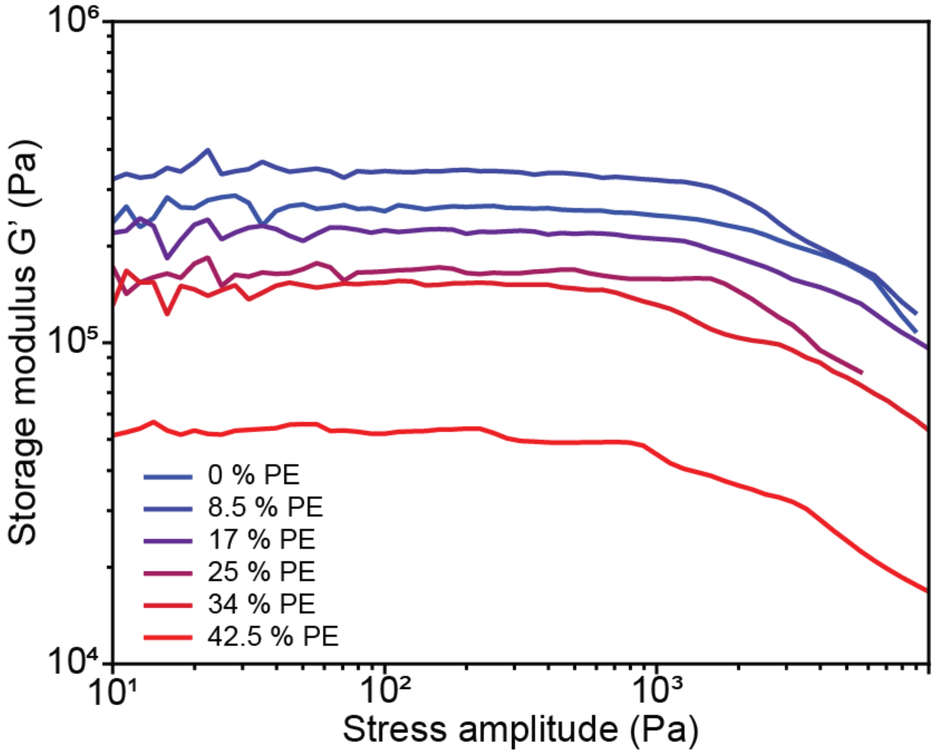

Figure S5. Rheological analysis of the different nanocomposites formulation. The storage modulus versus the oscillation stress amplitude for different PE contents in CNT-PE-SEBS composites is shown. The addition of PE seems to help to decrease both the storage modulus plateau and apparent yield stress. This is thought to be due to the semi-crystalline nature of PE which might help to initiate the flow in these composites with pronounced melt elasticity.

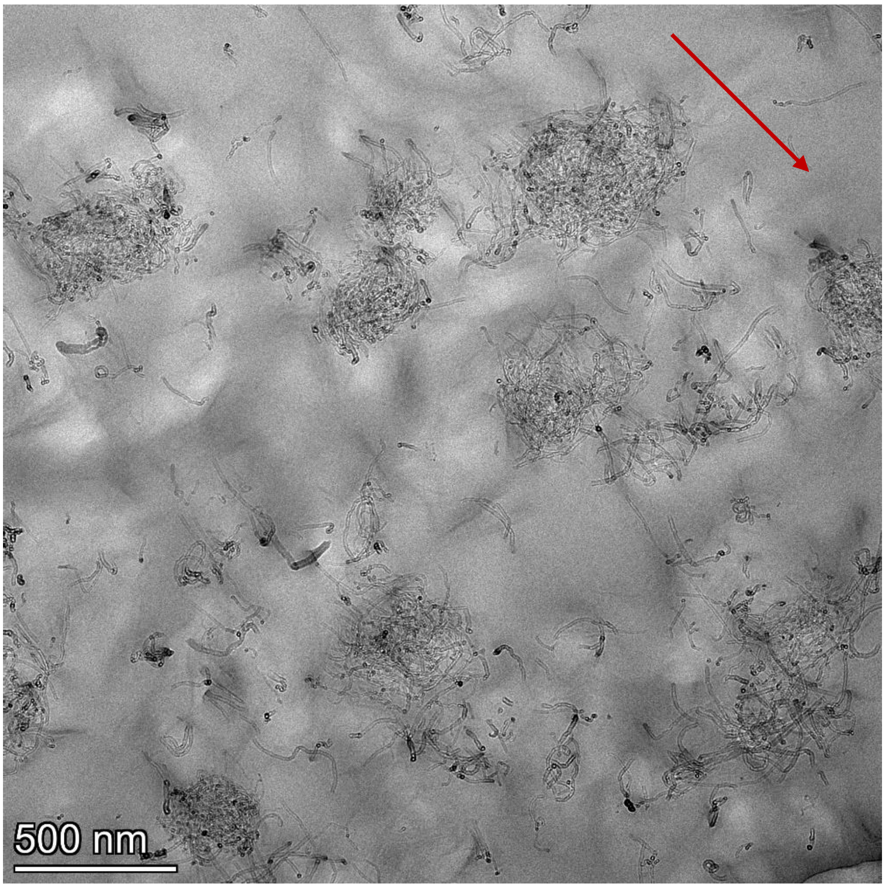

Figure S6. Transmission electron micrograph of 7.5 wt. % CNT- 42.5 wt. % PE-SEBS nanocomposite after thermal drawing. The arrow indicates the drawing direction.

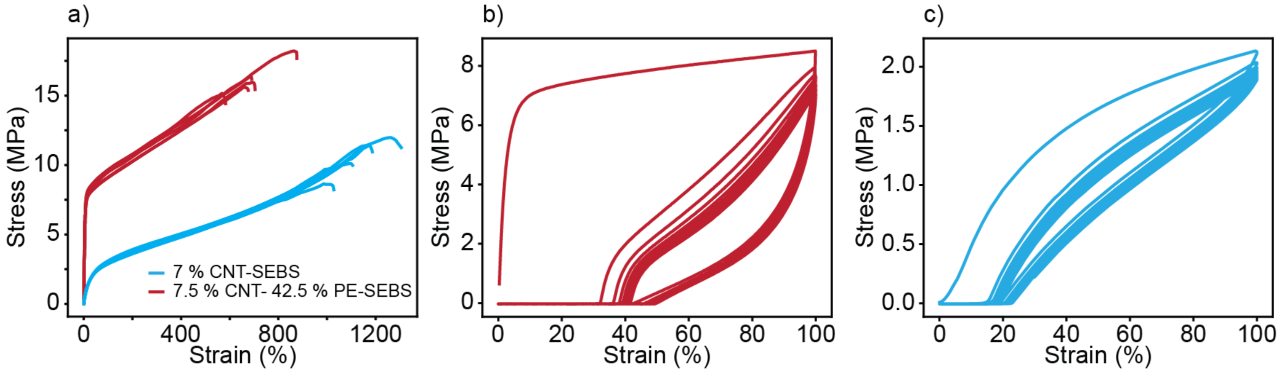

Figure S7. Characterization of the mechanical properties of the pure composite films. (a) Static tensile test for 7 % CNT-SEBS (blue) and 7.5 % CNT- 42.5 % PE-SEBS (red) composites. The addition of PE leads to a stiffer and less ductile material. (b) Cyclic tensile test of 7.5 % CNT- 42.5 % PE-SEBS (n_{cycles} = 10). (c) Cyclic tensile test of 7 % CNT-SEBS (n_{cycles} = 10).

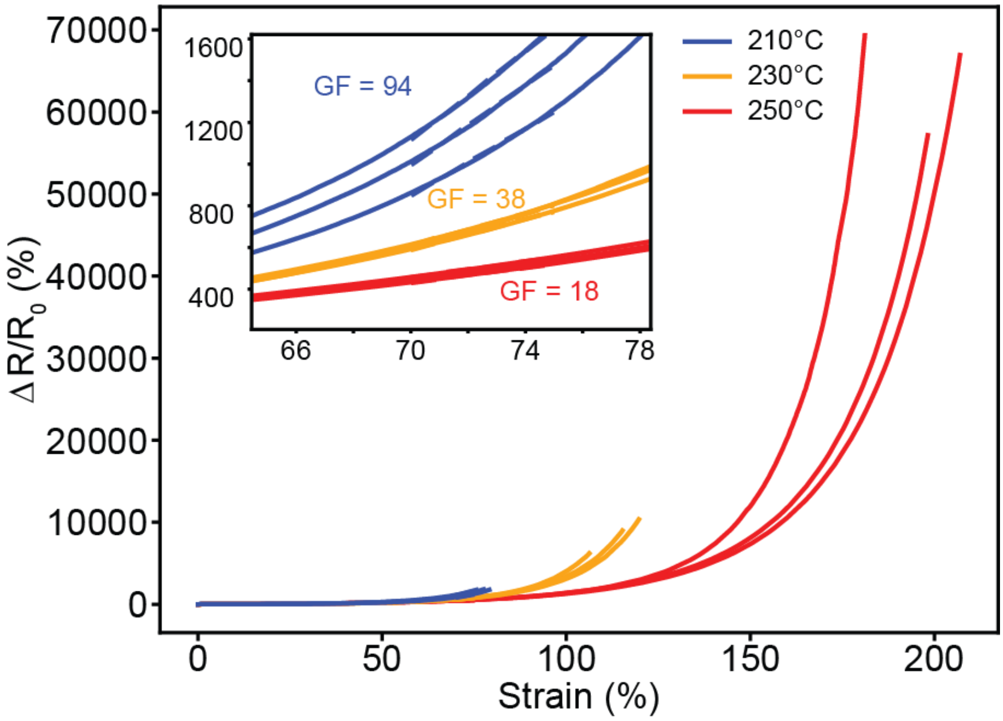

Figure S8. Gauge factor estimation. To compare the piezoresistivity of the nanocomposite processed at different drawing temperatures, a linear regression was performed around 70 % strain (dashed line). The slope reflects the gauge factor for this deformation state. Three samples were characterized to determine a mean value for the different drawing temperatures. The fibers were let one hour in the clamps before each test to enable material relaxation and remove any dependence of the resistance value on the strain history.

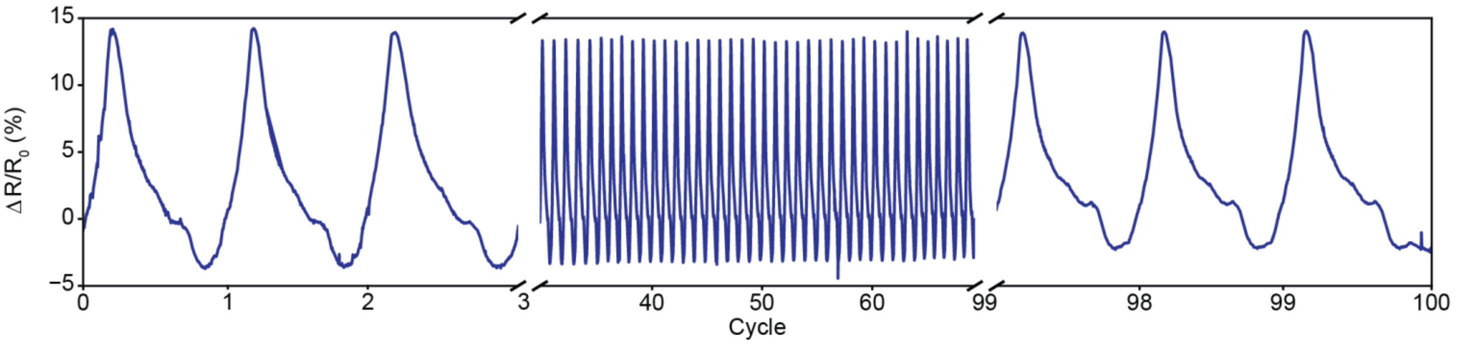

Figure S9. Fiber's response to cyclic bending deformation (n_{cycles} = 100). The relative resistance change of one electrode only is presented. For each cycle the angle is ramped from -90° to 90°. Consequently, the nanocomposite electrode experienced both a compression and a tension phase. The fiber shows a reproducible behavior for at least 100 cycles.

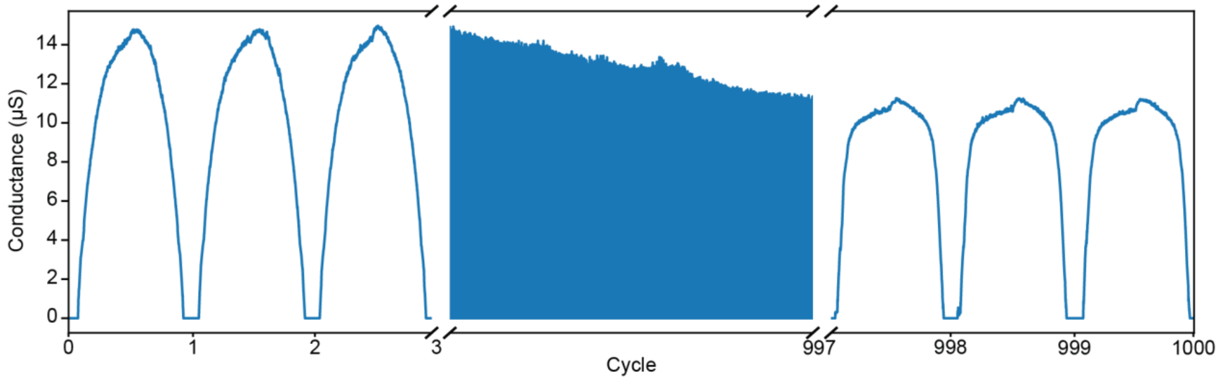

Figure S10. Fiber's response to cyclic compression test (n_{cycles} = 1000). The force is ramped from 0 to 15N. A decay is observed, and the peak of the last cycles represents 76 % of the initial response (first cycles). This might be caused by the viscoelastic nature of the fiber.

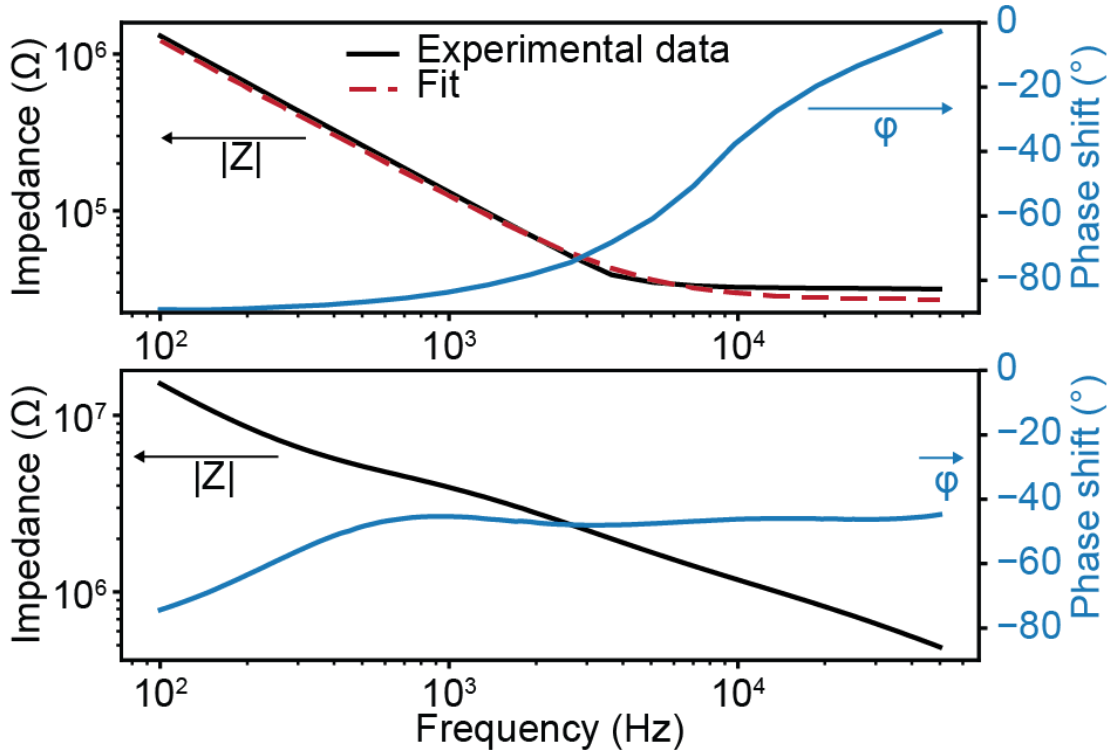

Figure S11. Frequency sweep experiment of a preform (top) and a fiber (bottom) obtained after thermal drawing. For the preform, by assuming a RC serie model (dotted line) the capacitance can be approximated to 1.3nF. After thermal drawing, due to the resistance increase of the nanocomposite electrodes, the fiber response doesn't follow the simple proposed model and the capacitance cannot be extracted.

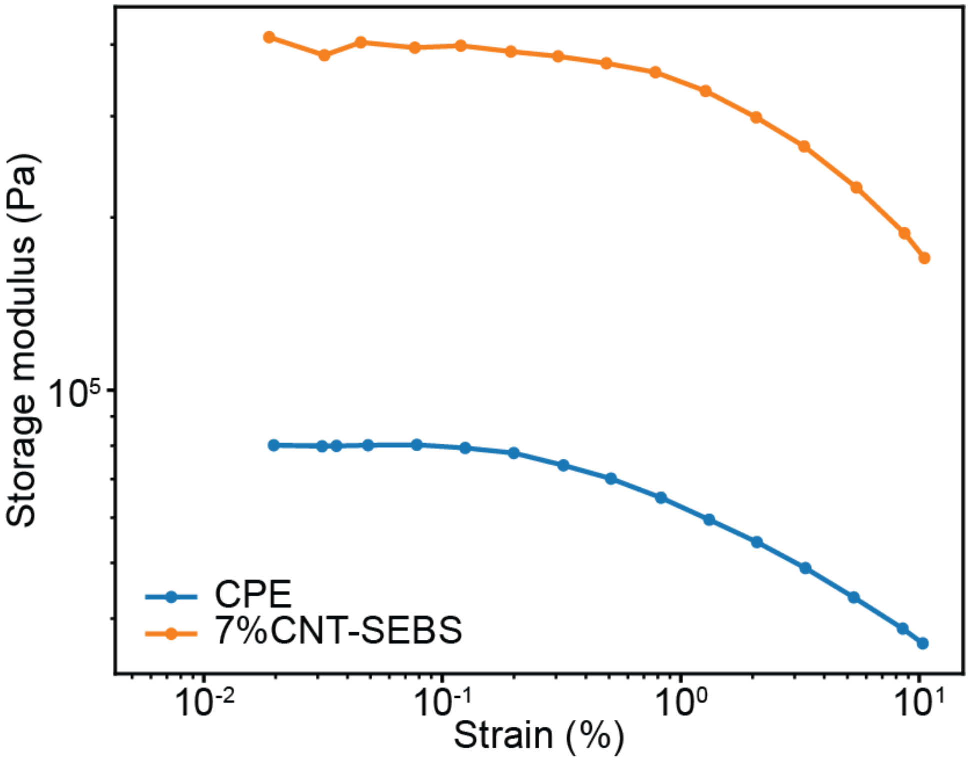

Figure S12. Strain sweep experiments. The critical strain level above which non-linear material's behavior is observed is approximately 1 % for the studied composite systems. Therefore, for further rheological characterizations, strain levels ≤ 1 % are chosen.
